# Exploratory use of docetaxel loaded acid-prepared mesoporous spheres for the treatment of malignant melanoma

**DOI:** 10.1186/s12645-015-0009-y

**Published:** 2015-01-24

**Authors:** Sameer Kaiser, Maximilian B MacPherson, Ted A James, Albert Emery, Page Spiess, Albert van der Vliet, Christopher C Landry, Arti Shukla

**Affiliations:** Department of Surgery, Danbury Hospital, Danbury, CT USA; Department of Pathology, University of Vermont, Burlington, VT USA; Division of Surgical Oncology, Department of Surgery, University of Vermont, 89 Beaumont Ave, Burlington, VT 05405 USA; University of Vermont College of Medicine, Burlington, VT USA; Department of Chemistry, University of Vermont, Burlington, VT USA

**Keywords:** Melanoma, Mesoporous, Silica, Microsphere, Nanoparticle, Docetaxel

## Abstract

**Introduction:**

Five year survival for metastatic melanoma (MM) is very low at <10%. Therapeutic options have been limited secondary to systemic toxicity. As a result there has been a growing movement towards developing targeted drug delivery models. Prior research of this group has demonstrated the effectiveness of acid-prepared mesoporous spheres (APMS-TEG) in delivering chemotherapeutic agents at a lower effective dose than systemic administration. This study aims to assess the ability of the previously developed APMS-TEG particles to deliver therapeutic doses of docetaxel for the treatment of melanoma.

**Methods:**

*In vitro* experiments were performed to assess docetaxel loading onto APMS-TEG particles and release kinetics. Toxicity experiments were performed using docetaxel and docetaxel loaded APMS-TEG. The effect on cell growth was assessed using the MelJuSo, UACC903, and WM1205 melanoma cell lines.

**Results:**

Docetaxel demonstrated statistically significant dose dependent reduction in growth of melanoma cells. In all three cell lines, doses of 1 nM were sufficient to produce statistically significant reduction in cell growth. Scanning electron micrographs demonstrate increased uptake of APMS-TEG particles by melanoma cells in the first 24 hours, with the majority within the first 4 hours. Unloaded APMS particles had no effect on the melanoma cells, demonstrating that the particles themselves are not toxic. APMS-TEG particles had a peak release of drug within the first hour, with equilibration thereafter. The 5, 10, and 20 nM loaded particles all had statistically significant reduction in cell growth than the control groups.

**Discussion:**

The high potency against melanoma cells makes docetaxel a suitable choice for loading into APMS-TEG particles. Docetaxel loaded APMS-TEG particles demonstrate significant activity against malignant melanoma and thus offer an innovative approach to the treatment of metastatic melanoma.

## Background

The incidence of melanoma has been steadily increasing over the past few decades. In the Caucasian population, the rate has tripled over the past 20 years [1]. Melanoma has become the 6^th^ most common cancer in the US, with an estimated 76,250 new cases in 2012 [2]. The true incidence however, may be even higher given underreporting of outpatient managed cases to cancer registries [3]. While melanoma accounts for only 4% of all skin cancers, it is responsible for more than 74% of all skin cancer related deaths [1].

Five year survival for those with metastatic melanoma (MM), 2-5% of melanoma diagnoses, is very low at <10% [4,5]. Therapeutic options for metastatic disease have also been limited. The mainstays of adjuvant therapy consist of dacarbazine and high dose interleukin-2 [5-7]. Chemotherapeutic drugs examined have included docetaxel (DOC), paclitaxel, temozolomide (TMZ), vinorelbine, and others [4-6]. Despite the number of chemotherapeutics that have activity against melanoma, these agents have had limited clinical use due to their systemic toxicities. The past few years have seen the arrival of ipilimumab and vemurafenib, which have improved survival in metastatic melanoma [4,8]. However, even these agents have limitations in their applicability due to their toxicity profiles.

As with other metastatic diseases, there has been a growing movement towards developing targeted drug delivery models in order to reduce systemic toxicity and enhance tumor toxicity. Docetaxel, which binds and stabilizes microtubules leading to mitotic arrest and eventually apoptosis, shows promise as an agent for targeted drug delivery due to its potent activity against melanoma. To date, there has been work from only two groups exploring the use of nanoparticles and nanomicelles to deliver docetaxel for MM [9,10]. Zheng, et al. created a polymer based nanoparticle as a delivery vehicle for DOC [9]. Though they show efficacy in killing melanoma cells, the particles are limited as they can only be administered via intratumoral injection. Ma, et al. describe preliminary work using nanomicelles loaded with chemotherapeutic agents, including DOC [10].

Nanoparticles show promise for drug delivery due to their small size, but carry the risk of systemic toxicity as well. By virtue of their size, these particles can enter and disrupt functioning of cellular organelles [11]. In order to address the issue of systemic toxicities, our prior research has focused on the development of a micro particle (1 – 3 μm diameter) that can be utilized for targeted drug delivery due to its porous nature [11]. These acid-prepared mesoporous spheres tagged with tetra ethylene glycol (APMS-TEG or APMS) have been proven to deliver chemotherapeutic agents to tumor cells at a lower effective dose than systemic administration [12]. Additionally, these particles have been demonstrated to show minimal systemic toxicity [11,12]. This study aims to assess the ability of the previously developed APMS-TEG particles to deliver therapeutic doses of DOC for the treatment of melanoma.

## Methods

### Human melanoma cell lines and reagents

Three human malignant melanoma cell lines, MelJuSo, UACC903, and WM1205, were obtained from the University of Vermont. All cells were maintained in 50:50 Dulbecco’s Modified Eagle Medium: Nutrient Mixture F-12 (DMEM/F12) containing 5% fetal bovine serum (FBS) and supplemented with penicillin (50 units/mL), streptomycin (100 μg/mL), hydrocortisone (100 μg/mL), insulin (2.5 μg/mL), transferrin (2.5 μg/mL), and selenium (2.5 μg/mL), and incubated at 37°C in 5% CO_2_. Cells were passaged weekly at a 1:10 ratio in to new flasks. Docetaxel (DOC) was obtained from Sigma (St Louis, MO.)

### Treatment of melanoma cells with docetaxel for assessment of cell growth

Cells were plated into 12 well plates at a density of 25,000 cells per well in 0.5 mL of medium described above and allowed to adhere for 2 – 3 hours before treatment. Varying doses of DOC (with equal total amounts of dimethyl sulfoxide (DMSO), the solvent control) were made up in fresh media and 0.5 mL was added to cells already plated to achieve the desired final concentrations of DOC. At 24, 48 and 72 h, cells were trypsinized, collected, and counted with a heamocytometer to determine average total cell numbers remaining at each time point for each dose.

### Scanning Electron Microscopy (SEM) of APMS-TEG interacting with MelJuSo melanoma cells

Acid prepared mesoporous spheres (APMS) tagged with tetra ethylene glycol (TEG) (APMS-TEG complex) were synthesized by Dr. Christopher Landry at the University of Vermont as previously described [11]. MelJuSo cells were grown to confluence on thermonox plastic coverslips (Nalge Nunc International, Naperville, IL) and were administered APMS-TEG particles (0.1 mg/mL). At 1, 4 and 24 hours, coverslips were fixed and prepared for SEM analysis as done previously [11]. In summary, coverslips were washed 2× for 5 minutes with 0.1 M Millonig’s phosphate buffer (pH 7.2), then fixed in 1:1 H_2_O dilution of Karnovsky’s fixative (2.5% glutaraldehyde, 1% paraformaldehyde) at 4°C for 45 minutes. Samples were then washed with Millonig’s phosphate buffer (pH 7.2), and post-fixed in osmium tetroxide (OsO_4_) at 4°C for 30–45 minutes. Samples were then dehydrated in graded ethanols, from 35% to 100%. Samples were critical point dried using liquid CO_2_ as the transition fluid in a Samdri PVT-3B critical point dryer (Tousimis Research Corporation, Rockville, MD). Specimens were mounted on aluminum specimen stubs using conductive graphite paint and allowed to dry, and were sputter-coated for 4–5 min with gold and palladium in a Polaron sputter coater (Model 5100; Quorum Technologies, Guelph, ON, Canada). Cells and APMS-TEG particles were then imaged on a JSM 6060 scanning electron microscope (JEOL USA, Inc., Peabody, MA) [11].

### Melanoma cell growth in response to docetaxel loaded APMS micro particles

APMS particles (2 mg) were incubated with increasing concentrations of docetaxel in 5 mL sterile distilled water for 24 – 72 h at 4°C on a rocking platform. APMS particles were then spun down to remove unloaded docetaxel still in solution, washed in water, spun down again and resuspended in 5 mL of fresh media. A 0.5 mL suspension of the loaded particles in media of each docetaxel concentration were then added to respective wells of a 12 well plate containing previously plated cells as described above. Controls used were DMSO alone, APMS (0.2 mg/mL) and 1 nM unloaded docetaxel. Concentrations of docetaxel indicated in these experiments represent the concentration of docetaxel in the loading solution and thus the maximal concentration of DOC that can be released if there is complete uptake and release from the APMS-TEG particles. Cells were collected at 24, 48, and 72 hours and total cell numbers were counted as described above.

### Release kinetics of docetaxel loaded APMS-TEG micro-particles

APMS particles (2 mg) were incubated with increasing concentrations of docetaxel in 5 mL sterile distilled water for 48 hours at 4°C on a rocking platform. APMS-TEG particles were then spun down with supernatant collected to quantitate the amount of unloaded docetaxel still in solution (unloaded). The particles were washed in water, spun down again, with the supernatant collected to quantitate the amount of DOC lost with washing (wash). Particles were resuspended in 10 mL of fresh media. A 1 mL suspension of the loaded particles in media of each docetaxel concentration was then added to respective wells of a 12 well plate. The control used was APMS (0.2 mg/mL) with no docetaxel. Concentrations of docetaxel indicated in these experiments represent the concentration of docetaxel in the loading solution and thus the maximal concentration of DOC released if there is complete uptake and release from the APMS-TEG particles. Media samples were collected at 1, 2, 4, 8, 24, and 48 hours.

### High performance liquid chromatography for detection of docetaxel

As per the protocol developed by Andersen, et al., a 3 μm Purospher STAR RP-18e (3×125 mm) column from EMD Millipore (Darmstadt, Germany) was obtained and maintained at 55°C [13]. Mobile phase consisted of 20 mM dibasic potassium phosphate buffer (pH 3): acetonitrile (57.5:42.5 v/v) run at 0.8 mL/min. Docetaxel peaks were seen between 7 – 8 minutes using a UV detector set to 227 nm. Fresh buffer and standards were prepared daily.

### Statistics

Statistical analysis was performed using GraphPad Prism 5.03/6.00. All data was analyzed by one way ANOVA followed by the Newman-Keuls Multiple Comparison Test or a student’s *t* test where indicated. Data with p <0.05 were determined to be significant.

## Results

**Figure 1:**
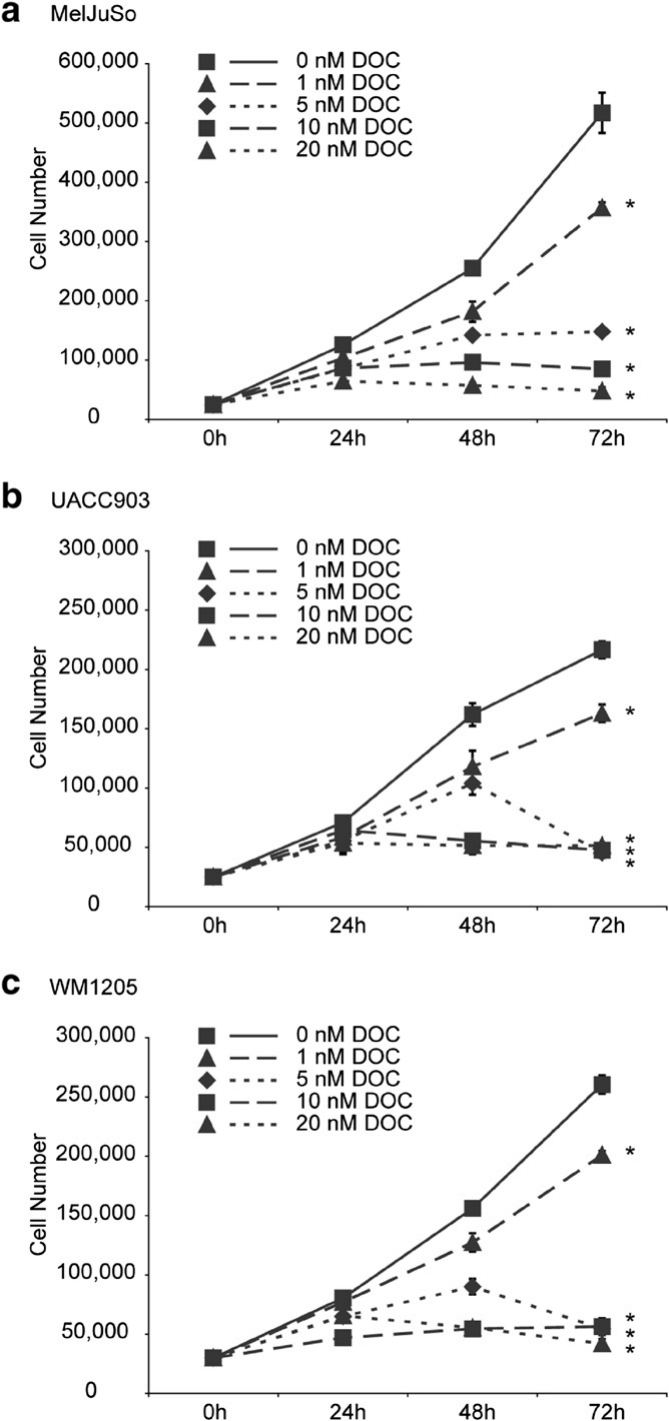
**Dose response of docetaxel (DOC) on the growth of 3 melanoma cell lines (a – c).** *, significant (p < 0.05) from 0 nM at 72 h.

Docetaxel was chosen as the model agent based on prior studies demonstrating melanoma cell death at doses in the nanomolar range [9]. DOC demonstrated statistically significant dose dependent reduction in growth of melanoma cells (Figure 1). In all three cell lines, doses of 1 nM were sufficient to reduce melanoma cell growth, which was statistically significant. A stronger and more uniform effect was observed at doses between 5 and 20 nM.

**Figure 2:**
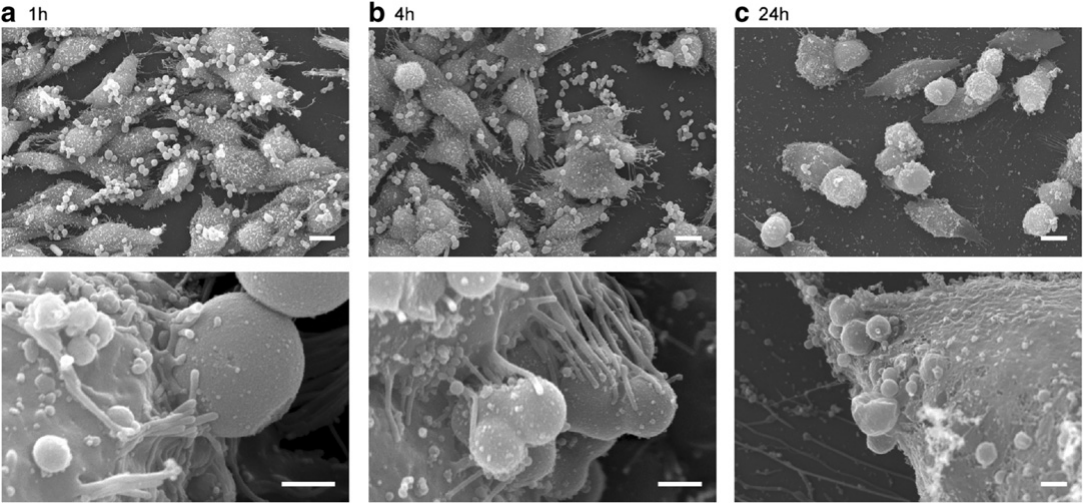
**Scanning electron micrograph showing MelJuSo melanoma cell uptake of APMS-TEG at different time points at two magnifications.** **a)** at one hour, **b)** at 4 hours and **c)** at 24 hours. Scale bar = 10 μm top row, 1 μm bottom row.

APMS-TEG particles were successfully prepared using the previously described technique. [11] Particles were administered to melanoma cells and examined under a scanning electron microscope (SEM) at varying time points (Figure 2). The SEM images demonstrate increased uptake of APMS-TEG particles by the melanoma cells over a 24 hour time course. Much of the particles are taken in within the first 4 hours, with little remaining in the extracellular space by 24 hours.

**Figure 3:**
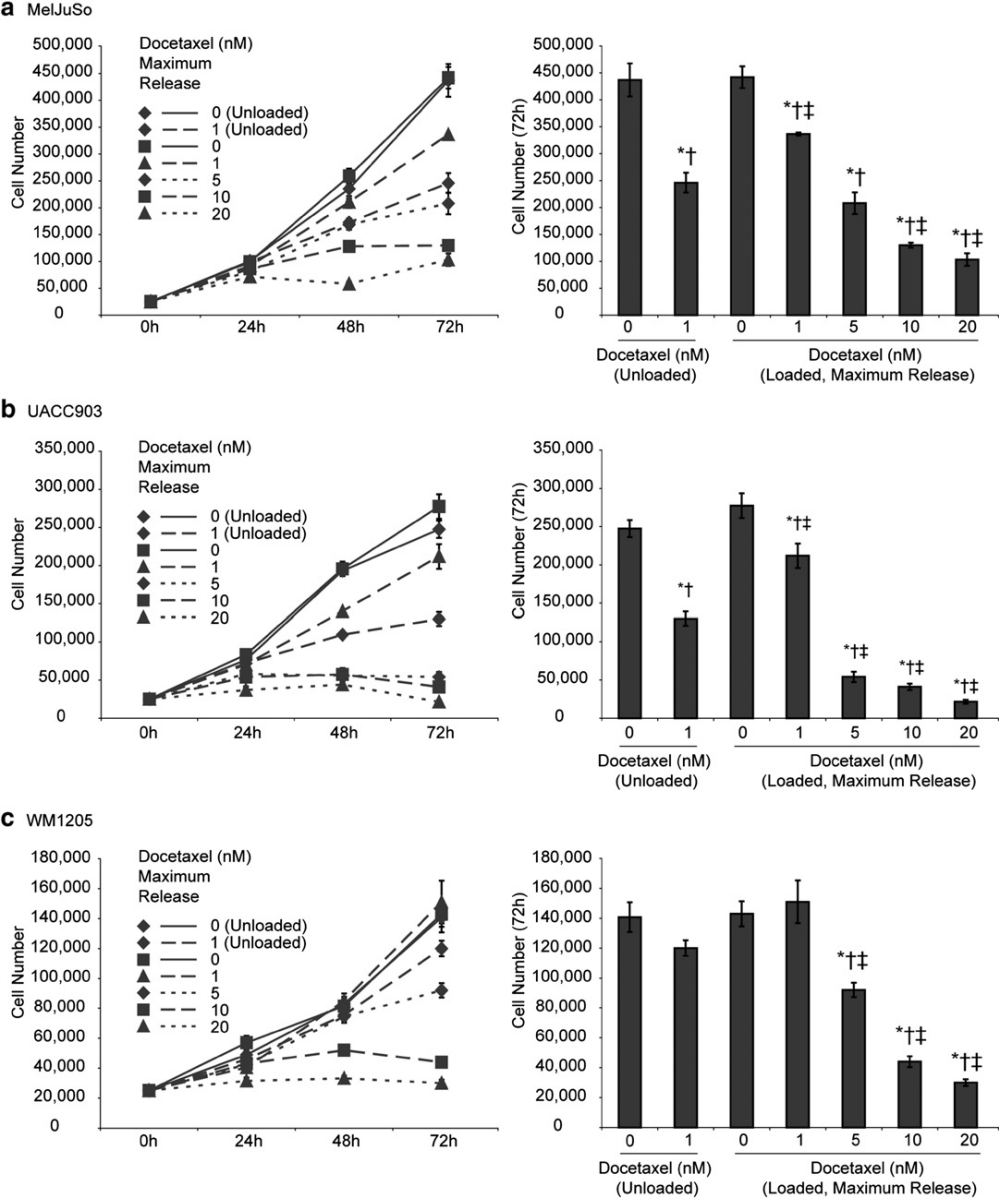
**Growth curves of melanoma cell lines.** **(a – c)** Left: Growth curves of three melanoma cell lines using APMS-TEG loaded with different concentrations of DOC. Right: Cell counts at 72 hours. *, significant (p < 0.05) from 0 nM (unloaded, negative control). †, significant (p < 0.05) from 0 nM (loaded in APMS-TEG, negative particle control). ‡, significant (p < 0.05) from 1 nM (unloaded, positive DOC control).

Varying concentrations of DOC were loaded into the APMS-TEG particles as described above and compared to directly administering a 1 nM dose of DOC and to particles with no DOC loaded. The first thing noted is that the unloaded particle has no effect on the melanoma cells, demonstrating that the particles themselves are not toxic (Figure 3). Particles loaded with higher concentrations of DOC achieved higher reductions of cell growth in all cell lines, similar to the results seen with unloaded DOC (Figure 1). The only exception to this is for the 1 nM loaded particles. These particles had a smaller effect than directly giving 1 nM of DOC (Figure 3, right panel). Though the loaded 1 nM particles had a smaller effect than the unloaded dose, the reduction in cell growth was still statistically significant in both the MelJuSo and UACC903 cell lines. The 5, 10, and 20 nM loaded particles all had statistically significant higher reduction in cell growth than any of the control groups.

**Figure 4:**
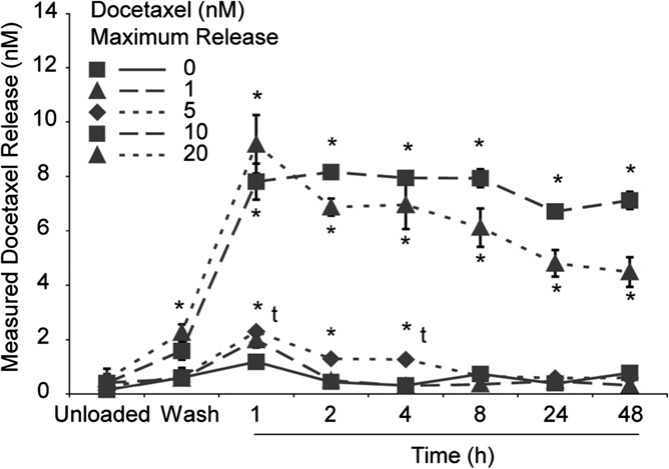
**DOC loaded APMS-TEG release kinetics.** *, significant (p < 0.05) from 0 nM at the same time point. t, significant (p < 0.05) by *t* test. All other significance was determined by one way ANOVA and a Newman-Keuls test for pairwise comparisons.

Analysis of the APMS-TEG particle loading and release kinetics demonstrates that essentially all the DOC is loaded into the particles after mixing (Figure 4, unloaded). Additionally, a minimal amount is lost in the wash step. In the 1 nM loaded particle, the peak concentration is 2 nM and diminishes after the first hour. For the remaining particles, all had statistically significant higher levels of DOC released into solution. All the particles have a peak release of drug within the first hour, with equilibration thereafter.

## Discussion

Our study demonstrated that DOC loaded APMS-TEG particles significantly reduce the growth of melanoma. The high potency against melanoma cells makes DOC a suitable choice for loading into APMS-TEG particles. In addition, scanning electron microscopy demonstrates that these particles are readily taken up by melanoma cells in a relatively short time frame.

When we load APMS-TEG particles with docetaxel, we see clear evidence of dose dependent reduction in cell growth. The particles do not demonstrate saturation with drug at doses as high as 20 nM. We see peak release of drug within 1 hour and this is sustained thereafter. The fact that we do not see all of the drug released in the 20 nM loaded particle is potentially a result of particles reaching equilibrium with the media it is suspended in. However, further studies are required to fully elucidate the release kinetics of DOC from the particles.

The toxicity profile of the APMS-TEG particles and mesoporous silica in general, is favorable when examining reviews of the existing literature. Nanoparticle formulations of mesoporous silica have been shown to be non-toxic with no evidence of inflammation in rat tendon, kidney, heart, and liver over 30 days after injection [14]. It can be safely administered subcutaneously, intravenously, and via intra-peritoneal routes with good bioavailability [15]. Mesoporous silica does not have carcinogenic potential [16]. These APMS-TEG particles have previously been shown to be non-immunogenic and non-toxic [11].

At this juncture, future directions for this model are twofold. The first goal is to move into animal models to validate *in-vivo* activity against melanoma cells. The second will be to characterize the pharmacokinetics and pharmacodynamics of the particle. This will include attaching functional moieties to the particle that will allow for targeted delivery of the particles to melanoma cells.

## Conclusions

APMS-TEG particles have previously demonstrated effectiveness at delivering chemotherapeutic drugs for other cancers, notably mesothelioma [11]. These early studies demonstrate that APMS-TEG particles can be used to deliver chemotherapeutic agents which could be used for the treatment of melanoma. Because these particles have been shown to be non-toxic and do not elicit an immune response, the problem of systemic toxicity associated with nanoparticle based delivery systems is reduced. Docetaxel loaded APMS-TEG particles demonstrate significant activity against malignant melanoma and thus offer a novel approach to the treatment of the disease.

## Abbreviations

MM: Metastatic melanoma
DOC: Docetaxel
TMZ: Temozolomide
APMS-TEG: Acid prepared mesoporous spheres tagged with tetra ethylene glycol
SEM: Scanning electron microscope(y)
